# Abdominal Surgery

**Published:** 1844-01-27

**Authors:** 


### ABDOMINAL SURGERY.

[On this interesting subject we have much pleasure in laying before our readers the following communication from Professor Dunglison.]

To the Editor of the Medical Examiner.

My dear Sir :—The recent interesting and successful cases of abdominal surgery, published by Dr. Clay and by Mr. Southam, of Manchester, Mr. Walne, of London, and Dr. John L. Atlee, of Lancaster, Pennsylvania, induce me to send you the "*Researches, Physiological and Pathological*," of Dr. Blundell, so long the able and eminent Lecturer on Physiology and Midwifery at the United Hospitals of St. Thomas and Guy, and well known, in this country, for his valuable contributions to obstetrical science. With a copy of this work, Dr. Blundell has recently favoured me, and has added manuscript notes, which have not been published, on some of the subjects, exhibiting his views at the present time. It will be admitted, that the facts and conclusions adduced by this distinguished individual have tended greatly to encourage surgeons in undertaking the bolder abdominal operations, of which we have recently seen the details, and at some of which he assisted. I am, my dear sir,

Very truly yours,

Robley Dunglison.

*Philadelphia*, 109 S. 10th St., Jan. 15, 1844.

In a paper read before the Medico-Chirurgical Society of London, in 1819, Dr. Blundell remarks :—"From various observations upon brutes, as well as my fellow-creatures, I cannot forbear imagining, that the risk of extensive inflammation, from local injury of the *peritoneum*, has been exaggerated, perhaps greatly. The high importance of this principle in surgery is too obvious to require a comment; already a sufficient number of observations has been accumulated to induce us to examine it with attention; and I may add, that it is one of those grand practical points which ought not to be decided by a few casual facts, much less by authorities however venerable; but, like every other principle of a sound philosophy, by various, deliberate, and unbiassed experiment and observation."

As the principle referred to in this extract did not excite so much attention as Dr. Blundell conceived it ought to do, he was afterwards led in his paper on "Abdominal Surgery," contained in the same work, to bring it again under the notice of the profession. This paper was read before the same Society, in the year 1823, but was not published. In it, after referring to various experiments upon animals, and to cases observed in this country and elsewhere, he thus concludes :*

" Such is the small collection of facts, *favourable and unfavourable*, which, with limited opportunities, I have been able gradually to accumulate in the course of the last five or six years; and which to me seem calculated to throw some additional light on the probable success of a more enlarged abdominal surgery. From these, few as they are, I feel conscious that no *certain* inference can yet be drawn, though *presumptive* inferences certainly may, and they seem to me to be the following:

1st. That smaller wounds of the peritoneum, as in tapping, hernia, &c., do not in general induce fatal peritonitis, or other destructive effects; and, therefore, that the common opinion, not perhaps found on paper, but frequently urged in conversation, and apparently operative in practice, I mean, that inflammation in a spot of the peritoneum, will almost invariably diffuse itself over the greater part of it, is probably unfounded in truth.

2d. That extensive divisions of the peritoneum are certainly not of necessity fatal, whether by inflammation or otherwise; and *probably* not generally so.

3d. That the womb, spleen, and ovaries may be taken away in the mode mentioned, certainly without of necessity destroying life, and *presumptively* without generally destroying it.

4th. That the womb, when developed from pregnancy, may be torn open; that the child may escape into the peritoneal sac, among the viscera; and that the mouth of the womb may be torn off, not, indeed, so far as these cases may be relied on, without great danger, but twice, in seven instances, without death.

5th. And generally, that the peritoneum and abdominal viscera, though very tender in the human body, will, without fatal consequences, bear more injury than, from their modes of practice, the British surgeons, especially, seem disposed to admit.

[Since the publication of this paper, many years ago, several additional cases of accidental division of the peritoneum have been communicated to me by different surgical friends, to whom they have occurred in the course of their own practice. In these cases, happening in men, women, or children, the abdomen has been variously laid open by stabbing, cutting, tearing, or goring ; and in almost every instance in which the intestines and other viscera have stood clear of grave injury as (stabbing cases, *perhaps*, excepted) they so frequently do, the patients seem, as a matter of course, to have recovered. When communicated by men of sense and probity, as falling under their own eyes, these cases almost always present the same features. 'Yes, the patient got well.' 'No, there was no dangerous peritonitis.' 'No, I never met with any other cases of the same kind in which the patients did not recover.' 'Yes, this is the only case, or those are the only cases, (as the fact may be,) which have occurred in my practice, and they recovered without difficulty.' 'That the intestines protruded—that they were replaced with difficulty—that it was necessary to enlarge the opening'—we are sometimes further told. Owing to the epidemic or a fear of peritonitis rife among the surgeons, these cases are generally mentioned as remarkable. 'It is very remarkable,' (meaning extraordinary,) 'it is very remarkable that the patient got well.' The truth, however, really is exactly the reverse. It would be very remarkable if the patient did *not* get well; for recovery in these cases is not the exception, but the rule. It is easy to verify all this by making the necessary inquiries. Truth is always the same.

JAMES BLUNDELL, M. D.]

6th. That all the above inferences, from observations on the human abdomen, are in unison with those drawn from observations on the rabbit, the one set of inferences mutually supporting the other; and in this we have a fact corroborative of the principle for which I have contended elsewhere, that observations on the brute and human subject, when made with caution, may, perhaps, be found more in correspondence with each other, than some surgeons are disposed, at present, to admit. A contrary opinion, so far as it is erroneous, must exert a very baneful influence upon the progress of surgery.

Whilst the body of facts which have reference to abdominal injuries remains so small, it would, no doubt, be the extreme of rashness, on such authority, to recommend to practice any operations as yet untried, or of rare performance, *unless, indeed, in those cases in which they secure the only remaining chance of life.* As, however, the facts related evidently create a suspicion, that a bolder abdominal surgery would not be unattended with success, I may be pardoned, perhaps, for endeavouring, on this occasion, to draw the notice of the profession to the following operations, all to appearance feasible, though, by no means, all of equal promise; stating distinctly, at the same time, that my design at present is to recommend them to *consideration* merely, and not to *practice*, except, as observed above, *in cases otherwise desperate.*

1st. *A division of both the Fallopian tubes, and even the removal of a small piece of them, so as to render them completely impervious—a fit addition, apparently, to the Cesarian operation, the danger of which it would scarcely increase.*—The effect of this operation would be to prevent subsequent impregnation, without, however, destroying the sexual propensities, or the menstrual action of the womb ; and, as many, besides Mr. Barlow's patient, have, on the Continent, recovered from the Cesarian operation, the possibility of a second need for it should, I think, by all means be precluded. In those cases, also, of contracted pelvis, in which, notwithstanding the excitement of parturition in the seventh month, it is still necessary to destroy the children, by opening the head, and reducing their size, in order to bring them down through the pelvis, I think it would not be amiss to adopt this operation in order to produce sterility. An opening, two fingers broad, might be made above the symphysis pubis, near the linea alba ; the fallopian tubes might be drawn up to this opening one after the other, and a piece of the tube might then be taken out. This operation, much less dangerous than a delivery by perforating the head when the pelvis is highly contracted, may, I think, be safely recommended.

2d. *The extirpation of the healthy ovaries.*—This operation, even granting it to be safe, can scarcely in any instance be necessary ; though it may be observ-

* [The manuscript notes, written within the last few months, are inclosed in brackets, and accompanied by the author's signature.]

ed, by the way, that it would probably be found an effectual remedy in the worst cases of dysmenorrhœa, and in bleeding from monthly determination on the inverted womb, where the extirpation of this organ was rejected.

3d. *The extirpation of the ovarian cyst in scirrhus, combined with dropsy or in simple dropsy.*—This operation will, I am persuaded, ultimately come into general use, and if the British surgeons will not patronize and perform it, the French and American surgeons will. If the dropsical cyst be large and of long standing, the removal will most probably be prevented by extensive adhesions; but if the cyst be small, containing (as in Nathan Smith's case) a few pints only, the adhesions will most probably be few and easily detached. It remains to be ascertained, *by observation*, to what extent adhesions in the abdomen may be cut through without danger to life.

4th. *The removal of a large circular piece of the cyst in ovarian dropsy, when the sack itself cannot be extirpated.*—As rupture of the ovary has cured the disease apparently, by laying the cyst open, and, perhaps, by inducing inflammation, advantage might be expected from this operation, at least as a palliative, though other cysts would no doubt, in many instances, gradually renew the disease.

5th. *The removal of the cancerous womb, when the ulceration first makes its appearance.*—Might not the womb be taken out above the symphysis pubis, or through the outlet of the pelvis? If above the symphysis pubis, might not the head of the vagina be tied up, and might not the ligature be conveyed by needle into the vagina, so as to hang out at the pudenda? All the parts about the cancerous womb, and the vagina among the rest, are in such a diseased state, that I expect little from this operation, unless early performed; and then, perhaps, Osianders' operation, of paring away the diseased surface of the ulcer, might be preferable, but I think the propriety of extirpating the womb in these cases ought certainly not to be lost sight of.

[Relying on the above principles, in the month of February, one thousand eight hundred and twenty-eight, in the presence of Dr. Elliotson and of three eminent surgeons, Bransby Cooper, Esq., Aston Key, Esq., and John Morgan, Esq., Dr. James Blundell (the author of this paper) removed the uterus by excision through the outlet of the pelvis, the organ being brought down, per vaginam, like the ovum of a six weeks' miscarriage. The case was one of bleeding disorganization of the os uteri, of a kind, under ordinary treatment, uniformly fatal. The patient recovered completely, becoming plump and well; but died on the 7th of February, one thousand eight hundred and twenty-nine, so that she lived twelve months, within a few days, after the performance of the operation; for it was on the 12th of February in the year preceding that the uterus was taken away. Death was clearly produced by enteritis following constipation; in part it may be occasioned, mechanically, from constriction and altered position of a portion of the bowel tied by adhesion, to which the inflammation resulting from the incision seemed to have given rise. On examining the body after death, Dr. Hodgkin found that the womb had been entirely removed, (see Medical Gazette, vol. iii., page 797, London,) and in the Museum of the Royal College of Physicians, London, it is now (1843) to be seen entire, having been presented to the Museum of the College by Dr. Blundell. The above operation was the second of four. In the other three the patients did not recover; they were all cases without other hope. The third and fourth operations were performed in consequence of the encouragement derived from the success of the second; and both of these were more neatly executed than the second and successful operation had been. Of these two last cases, however, it may be observed, that the disease was very far advanced. In the opinion of Dr. Blundell, the successful case derives its chief value from the proof which it affords of the solidity of the general principle on which it is grounded; namely, that the power of the abdomen to sustain operations of surgery is greater than, by British surgeons especially, has been supposed. In these four operations, the womb was removed by first, through the outlet of the pelvis, dividing, with a scalpel, the uterine attachments. The abdominal parietes were not touched. The two first of these operations, the second of which was the successful one, were the first of the kind performed in Great Britain. Under more favourable circumstances, better success may be anticipated.

JAMES BLUNDELL, M. D.]

6th. *Extirpation of the puerperal uterus.*—When the Cesarian operation is performed, or when a patient is evidently sinking after rupture of the womb, let it be remembered that the wound formed by the extirpation of the womb, and which might, probably, be much reduced in extent by drawing the parts together with a ligature, would merely take place of a more formidable wound—that, I mean, formed in the womb by the Cesarian operation, and which, by the operation here performed, would, together with the uterus, be taken completely out of the body. No operation, perhaps, can be more unpromising, shall I say more unjustifiable *in the present state of our knowledge?* but I thought it proper to mention it. Experiment on animals, rabbits for example, which have very large wombs, might be of use here; the inverted womb has been four times extirpated with success, when reduced to the original dimensions.

7th. Should the bladder give way into the peritoneal sac, and I have two preparations of this accident, why should we not lay open the abdomen, tie up the bladder, discharge the urine, and wash out the peritoneum thoroughly, by the injection of warm water? This operation would secure a chance of life, if the urine had not been extravasated long, say above half an hour.

8th. Small openings, with callous edges, through the neck of the bladder into the vagina, are cured in France, (as I learn from Mr. Travers,) by the actual cautery. When the opening is large, it may probably be closed by ligature, without a bad symptom. Mr. Preston, one of my pupils, first suggested to me this operation.

9th. The injection of astringent into the peritoneum, or into ovarian cyst, has been proposed, in cases of dropsy, to check the exhalation. The experiments related give little encouragement to the trial of this operation, at least with the oak bark; or rather, in the present state of our knowledge, they render it altogether unjustifiable.

10th. In cases of strongly characterized introsusception, though there never, perhaps, can be demonstrative proof of the disease, why should we not make an opening into the peritoneum, when every other remedy has failed, and gently pass the small intestines, fold by fold, through the fingers? In the dog and rabbit, (the latter animal has a tender belly and a large mass of intestines,) I have repeatedly done this without occasioning death, or even producing extensive and dangerous inflammation.

11th. In the rabbit I have often tied an abdominal artery, and then carried the ligature out of the abdomen, at the point where the artery lay, by means of a broad pointed needle, instead of drawing the thread forth at the wound. In operating on the human body, would this expedient be advantageous should further experience lead us to wish the ligature in all cases removed? I have once or twice, weeks after operating, found the remains of a ligature, which had been cut short, lying in the middle of a sack of puriform matter, and to appearance laying the foundation of chronic disease.

[In conformity with the above principles, the enlarged ovary, during the twenty years which have elapsed since the publication of this paper, has now (A. D. 1843) been removed in several instances successfully; in some cases by shorter incisions, in others, by longer. The first operations by long incisions performed in Great Britain, were those of Lizars; the last operations of this kind, known to Dr. Blundell, are those of Walne, done with an admirable combination of caution and enterprize, and the first which, in London, have been performed with success. At Mr. Walne's operations, two in number, Dr. Blundell was present. The incision was made in the linea alba; the length was of ten or twelve inches. The ovaries were dropsical, weighed sixteen or seventeen pounds, (imperial weight,) and were removed entire, without tapping. There were no adhesions. The ligatures, several in number, formed a complete skein, which stretched from the side of the pelvis to the linea alba, where the ends hung forth through the wounds. These ligatures, of course, contained within the peritoneum, remained for several weeks before they separated and came away. There were ten threads in the first of these two cases, and eight in the second; and in this second case a short, but severe attack of phlegmasia dolens supervened. Notwithstanding the wound of ten or more inches, and the peritoneal setons, to say nothing of the phlegmasia, in neither case did diffused and dangerous peritonitis at any time occur.—These operations, commencing with those of Lizars, performed all of them, and they are now not few, since the publication of this and the annexed paper, are grounded, as Mr. Lizars, in relation to his own operations, has, with a noble frankness, stated, on those general principles which these two papers contain. Of these two papers, the first, (on a few Controverted Points respecting Generation,) read before the Medico-Chirurgical Society (now the Royal) in the May of 1819, was published in the year 1819 in the 10th volume of their Transactions; and the second, (the above paper on Abdominal Surgery,) read before the Society in 1823, four years after the former, was, as a publication in the Transactions, declined, so that it did not appear in print till the year 1825, the date of this volume. That in the course of ages numbers will hereafter be saved by the means here recommended, the use of surgery within the abdomen, there is full reason to hope; and surely this, to say nothing of those that have been preserved already, furnishes a sufficient answer to all the obloquy with which, from well natured but misdirected feeling, the author and the author's experiments have been assailed. "Strike, gentlemen, but hear! You, who would join the laugh at the Egyptians, which will you sacrifice, your women or your cats?"

"*πάταξον μὲν ἄκουσον δέ.*"

JAMES BLUNDELL, M. D.]

[It was our intention to notice the interesting case of Dr. Atlee, and also that of Mr. Southam, in the present number of the EXAMINER, but the length of the foregoing article compels us to defer doing so until the next, when we shall probably have space for the purpose.—ED.]

